# Integrated diagnosis and treatment: Endoscopic retrograde direct cholangioscopy addressed the challenges of type III perforations

**DOI:** 10.1055/a-2615-1597

**Published:** 2025-07-25

**Authors:** Shan-Shan Hu, Xiao-Gang Liu, Yun-Chao Yang, Jie Hou, Wei-Hui Liu

**Affiliations:** 1 Department of Gastroenterology and Hepatology, Sichuan Provincial Peopleʼs Hospital, School of Medicine, University of Electronic Science and Technology of China, Chengdu, China

The complication of endoscopic retrograde cholangiopancreatography (ERCP)-related perforation (EP) has increasingly drawn clinical concern 1 . According to the Stapfer classification criteria, there exists a diagnostic and therapeutic dilemma for Type III perforations 2 3 . This report introduces a novel endoscopic retrograde direct cholangioscopy (ERDC) technique developed by our team 4 5 and demonstrates its technology as an effective method for the early diagnosis and treatment of Type III ERCP-related perforations.

A patient was scheduled to undergo ERCP. During intubation, the guidewire exhibited abnormal morphology. To facilitate early detection of EP, direct intubation was performed using ERDC-assisted ductal cannulation. This approach allowed for direct visualization, revealing that the guidewire had entered the peritoneal cavity and identifying ruptures in both the bile duct and pancreatic duct caused by the instrumentation ( Fig. 1 ). Using ERDC, we successfully guided the guidewire through the pancreatic duct rupture and placed a pancreatic duct stent ( Fig. 2 ). Similarly, ERDC facilitated identification of the compressed and obstructed bile duct orifice, allowing for smooth guidewire insertion. Upon further manipulation of the choledochoscope, the common bile duct was found to be narrow and slender, necessitating bougie dilation ( Fig. 3 ). Subsequently, endoscopic suturing technology was employed to close a suspected perforation near the duodenal papilla ( Fig. 4 ). Finally, a drainage tube was inserted along the guidewire into the common bile duct for effective drainage ( Fig. 5 ). Postoperatively, the patient experienced no discomfort, and abdominal computed tomography revealed no evidence of pneumoperitoneum or fluid accumulation ( Video 1 ).

**Fig. 1:**
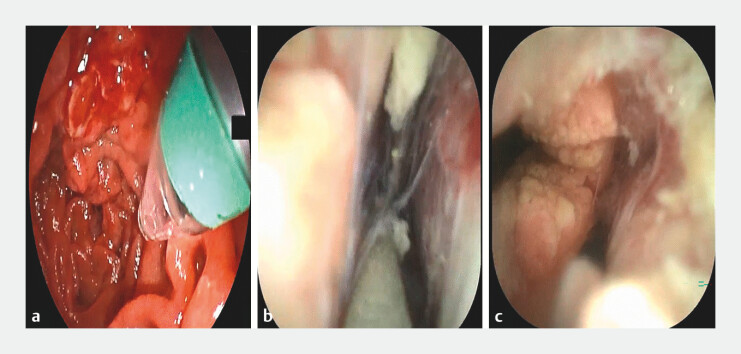
ERDC visualization of Type III EP: **a** Conical transparent cap mounted on the choledochoscope tip; **b** Guidewire visibly entering the abdominal cavity; **c** Identification of ruptures in the bile and pancreatic ducts. Abbreviation: EP, ERCP-related perforation.

**Fig. 2:**
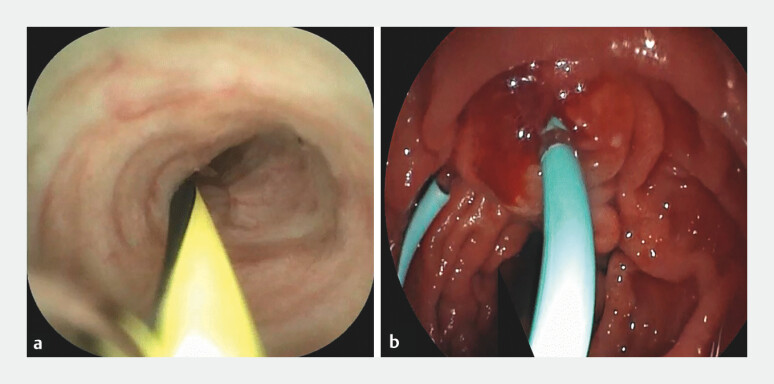
Super-selective pancreatic duct cannulation and stent placement: **a** Guidewire placement in the pancreatic duct under ERDC guidance; **b** Stent placement along the guidewire. Abbreviation: ERDC, endoscopic retrograde direct cholangioscopy.

**Fig. 3:**
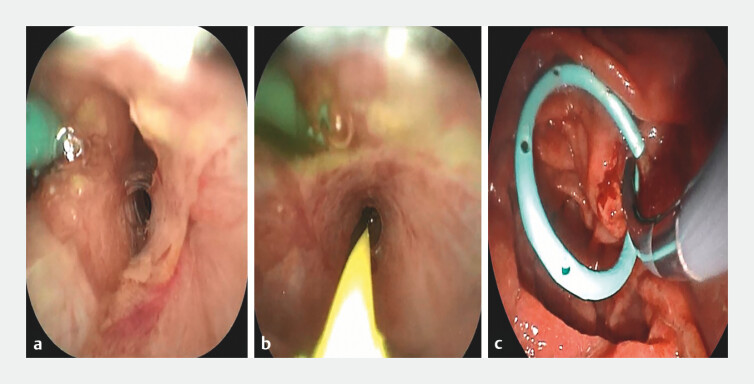
Super-selective bile duct cannulation and guidewire placement: **a** Guidewire placement in the bile duct under ERDC guidance; **b** Successful guidewire insertion; **c** Bougie dilation of the common bile duct. Abbreviation: ERDC, endoscopic retrograde direct cholangioscopy.

**Fig. 4:**
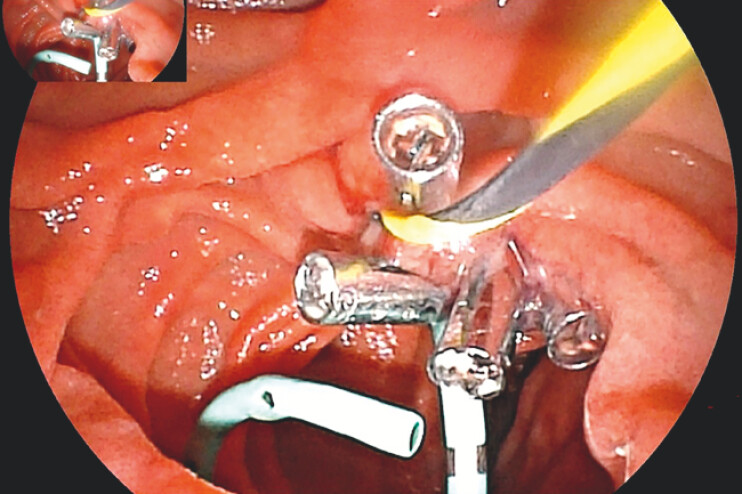
Endoscopic suturing technique.

**Fig. 5:**
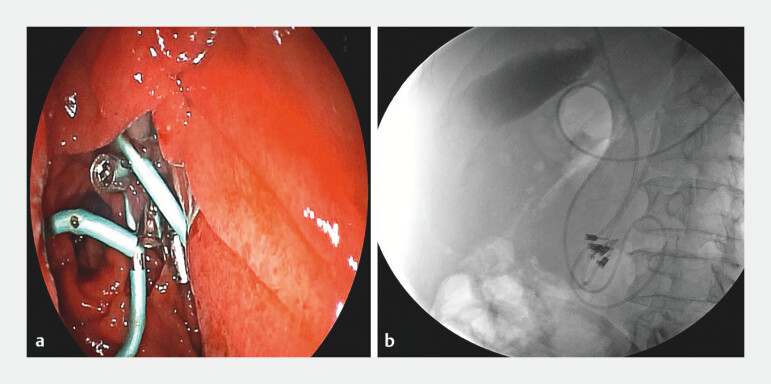
Placement of the biliary drainage tube: **a** Insertion of the drainage tube along the guidewire; **b** Fluoroscopic confirmation of correct placement of the drainage tube, stent, and metal clip, with no contrast agent leakage observed.

The visual capabilities of ERDC enabled the early identification of Type III EP. ERDC offers a direct and intuitive method for observing ruptures in the bile or pancreatic ducts.Video 1

In this case, the visual capabilities of ERDC enabled the early identification of Type III EP. In contrast to conventional ERCP, which depends on contrast agent diffusion for EP diagnosis and carries a risk of inducing peritoneal infection, ERDC offers a direct and intuitive method for observing ruptures in the bile or pancreatic ducts. This facilitates precise and selective intubation.

Endoscopy_UCTN_Code_TTT_1AR_2AK

## Notes

Endoscopy E-Videos https://eref.thieme.de/e-videos
*E-Videos* is an open access online section of the journal *Endoscopy* , reporting on interesting cases and new techniques in gastroenterological endoscopy. All papers include a high-quality video and are published with a Creative Commons CC-BY license. Endoscopy E-Videos qualify for HINARI discounts and waivers and eligibility is automatically checked during the submission process. We grant 100% waivers to articles whose corresponding authors are based in Group A countries and 50% waivers to those who are based in Group B countries as classified by Research4Life (see: https://www.research4life.org/access/eligibility/ ). This section has its own submission website at https://mc.manuscriptcentral.com/e-videos .
